# Surface Functionalization of Sugarcane-Bagasse-Derived Cellulose Nanocrystal for Pickering Emulsion Gel: Microstructural Properties and Stability Efficiency

**DOI:** 10.3390/gels9090734

**Published:** 2023-09-09

**Authors:** Shao Hui Teo, Yern Chee Ching, Mochamad Zakki Fahmi, Hwei Voon Lee

**Affiliations:** 1 Nanotechnology & Catalysis Research Center (NANOCAT), University of Malaya, Kuala Lumpur 50603, Malaysia; julianteoshaohui@um.edu.my; 2 Department of Chemical Engineering, Faculty of Engineering, University of Malaya, Kuala Lumpur 50603, Malaysia; chingyc@um.edu.my; 3 Sustainable Process Engineering Center (SPEC), Faculty of Engineering, University of Malaya, Kuala Lumpur 50603, Malaysia; 4 Department of Chemistry, Faculty of Science and Technology, Universitas Airlangga, Surabaya 60115, Indonesia

**Keywords:** biomass, nanocellulose, stearyltrimethylammonium chloride, surface modification, dispersion–adsorption, colloidal stability, rheology analysis

## Abstract

An environmentally friendly Pickering stabilizer was developed by upcycling sugarcane bagasse (SCB) into a cellulose nanocrystal (CNC), which was subjected to surface modification by using quaternary ammonium compound to enhance its amphiphilic characteristics. The changes in microstructural properties of modified cellulose nanocrystal (m-CNC), such as surface functional group, thermal stability, surface morphology, elemental composition, and particle size distribution were investigated. Results indicated the success of quaternary ammonium compound grafting with the presence of a trimethyl-alkyl chain on the cellulose structure, while the m-CNC preserves the needle-like nanoparticles in length of ~534 nm and width of ~20 nm. The colloidal profile of m-CNC-stabilized oil–water emulsion gels with different concentrations of m-CNC (1–5 wt%), and oil:water (O:W) ratios (3:7, 5:5, 7:3) were examined. The emulsion gel stability study indicated that the optimal concentration of m-CNC (3 wt%) was able to stabilize all the emulsion gels at different O:W ratios with an emulsion index of >80% for 3 months. It is the minimum concentration of m-CNC to form a robust colloidal network around the small oil droplets, leading to the formation of stable emulsion gels. The emulsion gel with O:W ratio (3:7) with 3 wt% of m-CNC rendered the best m-CNC–oil-droplets dispersion. The m-CNC effectively retained the size of oil droplets (<10 μm for 3 months storage) against coalescence and creaming by creating a steric barrier between the two immiscible phases. Furthermore, the emulsion gel exhibited the highest viscosity and storage modulus which was able to prevent creaming or sedimentation of the emulsion gels.

## 1. Introduction

The world’s population has reached 8 billion and is projected to rise to 10.9 billion (with 95% prediction interval of 9.4–12.7 billion) in the year 2100 [1]. This phenomenon has led to a surge in the need for food and gives rise to challenges of ensuring food security in the upcoming years. To keep up with the rising demands from the growing population, the production of both crops and livestock has increased significantly, which indirectly co-generate high amounts of agricultural wastes [2]. Agro-industrial waste is one of the several categories of agricultural wastes that has raised public awareness and threatened the sustainability of agricultural cultivation as its disposal and utilization are not efficiently managed [3]. Sugarcane bagasse (SCB) is one of the most abundant agro-industrial waste products generated from the sugar industry, as well as from ethanol manufacturing plants [4]. It is reported that approximately 180.73 million metric tons of SCB is produced worldwide annually and may increase to 221 million metric tons by the year 2024 [2]. Generally, the majority of the SCB are not utilized sustainably, but left to decompose naturally in landfill. Another way of disposal involves direct combustion of SCB for energy which yields carbon dioxide, organic matter oxidation products, smoke, and particulate matters that contribute to environmental emission pollution [5]. However, SCB is a type of lignocellulosic biomass mainly comprised of cellulose fibers (36.9 to 45.7%) that can be extracted out for a variety of uses [6].

Cellulose has been well-established for its versatile usage in various applications such as the manufacturing of paper, cardboard, and derivative products like rayon and cellophane [7]. Upcycling of abundant and renewable agro-industrial waste (like SCB) to cellulose will reduce SCB burning, while adding value to SCB. This can be beneficial not only to the environment but also financially to one’s country, building a circular economy and providing environmental protection.

In the last few decades, there has been an increase in research on nanocellulose as a non-toxic and environmentally friendly material. Nanocellulose shows remarkable properties (high aspect ratio, large specific area, low density, and high crystallinity) as well as the potential to be tailored by functionalization due to the numerous hydroxyl groups (-OH) present in the cellulose compound [8,9]. Nanocellulose has the ability to produce emulsions gels that can remain stable for several months under various extreme conditions, which may be attributed to steric and electrostatic effects [10]. In addition to that, properties like renewability, low toxicity, chemical stability, and biocompatibility also make them suitable for their use in emulsion stabilization [11]. Thus, nanocellulose has emerged as a promising bio-based Pickering stabilizer with potential for a wide range of liquid-, gel-, and cream-based formulations in the food and beverage, cosmetics, personal care, hygiene, pharmaceutical, and biomedical fields [12].

Nanocellulose has several advantages over the widely used conventional surfactants as an emulsifier. Firstly, nanocellulose can be synthesized from renewable sources (plant biomass) while surfactants are derived from fossil fuels, making nanocellulose a sustainable and renewable product. However, the low nanocellulose yield might become an issue in a large-scale production as it is dependent on many factors such as the type of raw material, biomass pre-treatment, and cellulose size reduction process, as well as the reaction parameters of each treatment stage. Secondly, most surfactants of synthetic origins like sodium dodecyl sulfate, Sorbitan esters, etc., were found to possess a high profile of toxicity and low biodegradability [13,14]. The conventional surfactants are capable of destroying aquatic microbial populations, harming aquatic life, reducing photochemical energy conversion efficiency of plants, and affecting the waste-water treatment process [15]. On the other hand, nanocellulose derived from lignocellulosic biomass has a low toxicity and high biocompatibility. In addition, a lower amount of nanocellulose is required to achieve the desired emulsion stability when compared to conventional surfactants as their stabilization mechanism differed and the nanocellulose has an extremely high surface area [16,17].

However, cellulosic materials are generally regarded as being more hydrophilic due to the numerous hydroxyl groups on their surface even though cellulose is believed to be slightly amphiphilic due to the crystalline organization at its elementary level before undergoing any treatment [18]. The many hydroxyl groups also cause the fibers to aggregate during processing, leading to poor dispersion and stability in water which limit its applicability [19]. Thus, various surface modification approaches have been developed for nanocellulose to make functional nanocellulose with a wide range of wettability properties and to expand their use as effective Pickering emulsifiers [20]. Additionally, the modification could prevent the agglomeration of nanocellulose particles and at the same time improve their dispersion in water, depending on their respective zeta potential measurements [19]. This can be achieved by imparting a hydrophobic molecule to the cellulose compound which can be done through chemical, physical, or even biological interactions [21]. Du Le et al. utilized the chemical method by modifying nanocellulose with octenyl succinic anhydride through esterification and displayed a static water contact angle of 80.2° [22]. At a high ionic strength (≥20 mM NaCl) and low pH (<4.0), the emulsion showed a prominent gel-like character which can be applied in the design of an emulsion system to target the delivery of bioactive compounds. Parajuli et al. modified nanocellulose using the physical method with several surfactants in a high ionic strength environment and studied their interfacial properties as well as the dodecane/brine emulsion stability. They found that the ionic strength, adsorption energy, wettability, and oil content affected the emulsion stability [23]. Chen et al., utilized ocetenyl succinic anhydride to modify cellulose nanocrystal (CNC) and was able to improve the emulsification performance of CNC and form a stable high internal phase Pickering emulsion. This was attributed to the enhanced hydrophobicity of CNC after the modification process. Not only that, the increase in hydrophobicity contributed to the increment of its zeta potential that could affect its stability in water [24].

There was limited study on the use of stearyltrimethylammonium chloride (STAC)-modified cellulose nanocrystal as a Pickering stabilizer. STAC is a type of quaternary ammonium compound used as cosmetic ingredients for the purpose of hair conditioning, surfactant cleansing, and antistatic products [25]. It possesses a hydrophilic head and hydrophobic tail, suitable to improve the hydrophobicity of CNC. A study by Saidane et al. reported that CNC modified by STAC successfully formed a stable water-in-hexadecane emulsion. Nonetheless, there is an absence of investigation confirming this observation, along with a depth of analysis concerning the rheology and microstructural profile of the emulsion samples [26]. Therefore, this study aimed to utilize SCB residue and convert it into a modified cellulose nanocrystal (m-CNC) using STAC, followed by evaluating its practicability as an effective Pickering stabilizer for emulsion gels. In this work, we employed the facile method to functionalize CNC through electrostatic attraction, which reduces solvent and chemical usage. The microstructural characteristics of m-CNC were examined in terms of surface functional group profile, thermal stability, surface morphology, elemental profile, and particle size distribution. In addition to that, the effectiveness of the m-CNC-stabilized emulsion gels (emulsion volume, microstructure of emulsion layer, and rheology profile) was investigated with different concentrations of m-CNC (1–5 wt%) and oil:water (O:W) ratios of 3:7, 5:5, 7:3. This study revealed the possibility of converting lignocellulosic biomass waste (SCB) into functionalized nanocellulose that can be used as an effective Pickering stabilizer for emulsion-gel-based applications.

## 2. Results and Discussion

### 2.1. Characterization of m-CNC Pickering Stabilizer

#### Physicochemical Properties of m-CNC-Based Pickering Stabilizer

The yield of product obtained from each respective process and the percentage yield of products from the raw material were calculated using Equations (1) and (2) (Table 1). An average of 41.01% yield of chemically purified cellulose (CPC) was obtained after the SCB pre-treatment process. The bleached fibers then underwent an acid hydrolysis process to convert the cellulose fibers into CNC. The percentage yield of the CNC was 37.81% and 15.51% with respect to the mass of dried CPC and raw SCB, respectively. The low yield obtained can be attributed to the removal of hemicellulose and lignin during the pre-treatment process as they comprised ~60% of the total composition of SCB. In addition, the amorphous region of the cellulose chains was hydrolyzed during the acid hydrolysis process, decreasing its percentage yield. Lastly, no loss of product was observed during the modification process.

**Figure 1:**
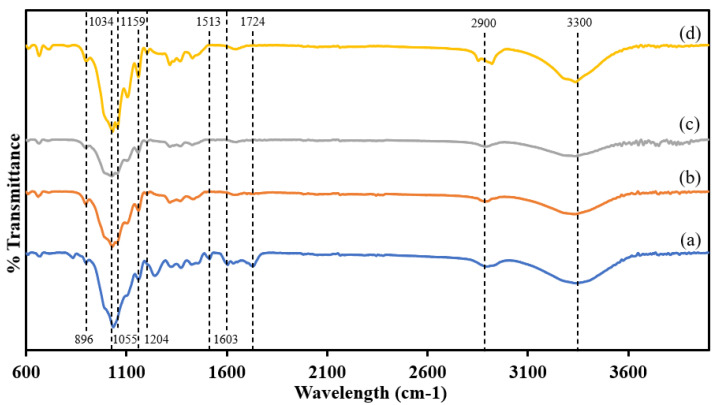
FTIR spectra of (**a**) raw SCB, (**b**) CPC, (**c**) CNC, and (**d**) m-CNC.

The changes in surface functional groups of SCB, CPC, CNC, and m-CNC after several treatments were analyzed using Fourier transform infrared spectroscopy (FTIR) as shown in Figure 1. Raw SCB shows the presence of stretching vibrations of the carboxyl group (-C=O) at the FTIR band of 1724 cm^−1^ [27]. Meanwhile, the peaks at 1603 and 1513 cm^−1^ (between ~1600 and 1500) were characterized as the in-plane symmetrical stretching of the aromatic ring (-C=C-). The disappearance of the above-mentioned FTIR peaks in CPC indicated that hemicellulose and lignin were completely extracted from the biomass complex [28,29,30].

The natural structures of CPC, CNC, and m-CNC are present in cellulose molecules; thus, the samples exhibited similar FTIR spectra with minimal changes. The presence of C-O anti-symmetric ring stretching was reflected by the 1159 cm^−1^ band, while cellulose β-glucosidic ether linkages were shown at 1034 and 896 cm^−1^ of the FTIR peaks [28,31]. The cellulose-based samples show high intensities for the mentioned FTIR peaks as compared to SCB, due to higher purities of cellulose content [30,32,33].

The successful modification of m-CNC with STAC was confirmed with the presence of 2912 cm^−1^ and 2854 cm^−1^ that were attributed to the asymmetric and symmetric C-H stretching vibrations of the aliphatic methylene groups (-CH_2_) along the alkyl chains, respectively [34,35]. Furthermore, another two new peaks were observed at 1204 cm^−1^ and 1055 cm^−1^, corresponding to the stretching vibrations of the C-N bond.

**Figure 2:**
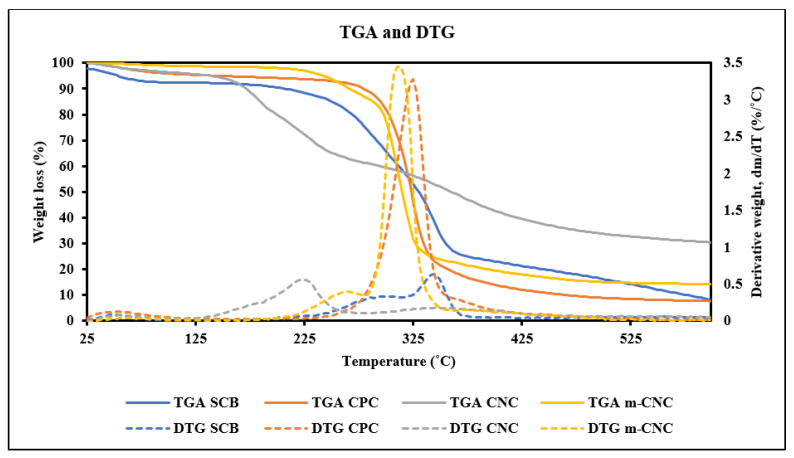
TGA and DTG curves of raw SCB, CPC, CNC, and m-CNC.

The thermal stability of the raw SCB fibers, CPC, CNC, and m-CNC were studied through thermogravimetric analysis (TGA). Figure 2 shows the TGA and derivative thermogravimetric analysis (DTG) graph of the samples. Most of the weight lost for all the samples occurred at the temperature range of 200–400 °C which was due to the thermal degradation of the cellulose compound. Their thermal stability profile was also observed to decrease in the order of SCB > CPC > m-CNC > CNC.

Degradation of raw SCB showed two DTG peaks indicating the breakdown of at least two compounds. The degradation of hemicellulose in SCB was discovered in the first peak with an onset degradation temperature of 277 °C. After the hemicellulose had been completely degraded, cellulose in SCB was observed to start degrading with an onset temperature of thermal degradation (T_on_) of 320 °C and maximum degradation temperature (T_max_) of 340 °C.

Cellulose is considered to be highly crystalline when compared to hemicellulose; therefore, it is more thermally stable and degrades at a higher temperature [31]. As expected, the CPC exhibited a higher T_on_ of 299 °C and T_max_ of 325 °C when compared to the raw SCB. The DTG displayed a sharp peak at 325 °C, showing the thermal degradation of cellulose, indicating the successful extraction of most of the lignin and hemicellulose.

However, the CNC exhibited lower thermal stability after acid hydrolysis treatment, which indicated that the cellulose structure degraded within a reduced temperature range (around 200–400 °C). This was due to the esterification of hydroxyl groups into sulfate groups (OSO_3_^−^) in the cellulose compound during the hydrolysis process [31]. The -OSO_3_^−^ present on the surface of nanocellulose provided a catalytic effect in the process of thermal degradation and lowered the activation energy of thermal degradation. The DTG curve showed two peaks with T_on_ of 192 °C and 306 °C and T_max_ of 226 °C, indicating the degradation of CNC occurs in two stages [36,37]. The first deterioration process observed was due to the degradation of sulfated nanocellulose while the second degradation process was attributed to the decomposition of un-sulfated nanocellulose.

The m-CNC with STAC surface modification was able to improve the thermal stability of CNC tremendously as observed in the TGA and DTG curve. The high thermal degradation temperature can be attributed to the protective effect of the large molecular STAC, which shielded the OSO_3_^−^ [35]. In addition, the DTG curve showed two shoulder/humps that were similar to CNC, but had a higher T_on_ of 235 °C and 289 °C and a higher T_max_ of 310 °C.

**Figure 3:**
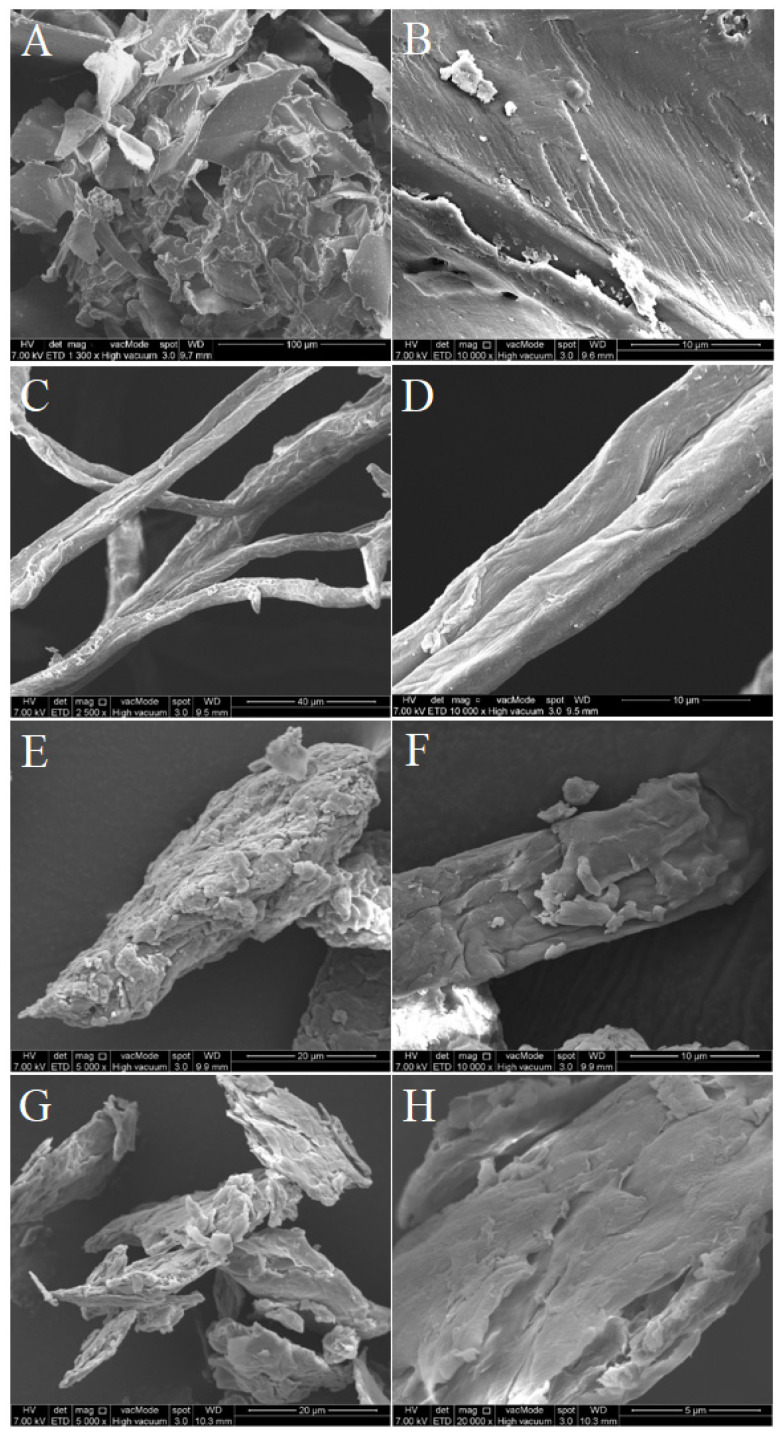
FESEM micrographs of (**A**,**B**) raw SCB, (**C**,**D**) CPC, (**E**,**F**) CNC, and (**G**,**H**) m-CNC.

The surface morphology of raw SCB, CPC, CNC, and m-CNC was analyzed using field emission scanning electron microscopy (FESEM) and could be observed in Figure 3. The raw SCB presented an irregular shape with size ranging from several hundred microns with low aspect ratio (Figure 3A). The raw SCB had a relatively smooth and uniform external cell tissue on its surface which consisted of the three main composition of lignocellulosic biomass (cellulose, hemicellulose, and lignin) [38]. The dimension of the CPC was reduced dramatically and measured with a width of several microns and a length of several hundred microns, indicating an increase in the aspect ratio (Figure 3C,D). The reduction in size can be explained by: (i) the removal of non-cellulosic compounds like hemicellulose and lignin from the raw material which caused the most decrement in size; (ii) defibrillation of the cellulose fibers into cellulose microfibrils via depolymerization. For both CNC and m-CNC, both samples displayed similar morphological characteristics with a bulk aggregate sized several microns (Figure 3E–H). During the drying process, the nanocellulose fibers were inclined to grow closer to each other as more water molecules evaporated [39]. This allowed the fibers to form hydrogen bonds between the existing hydroxyl groups in both lateral and longitudinal positions, and lastly aggregated into a 3D structure [40]. Hence, this could provide an explanation for the presence of aggregate-like structures displaying an uneven surface, which resulted from the self-agglomeration of nanocellulose.

Energy-dispersive X-ray spectroscopy (EDX) and CHNS analyses were performed on CPC, CNC, and m-CNC to determine their elemental composition. The weight and atomic composition of the analyzed element for each sample were summarized and tabulated in Table 2. It was found that the conversion of CPC to CNC did not impact the C and O content as their composition remained relatively the same. The acid hydrolysis process hydrolyzed the amorphous region of the cellulose chain while leaving the crystalline region intact. However, there was an increase in its sulfur composition from 0 to 0.55 wt% after the acid hydrolysis process. This was due to the negatively charged OSO_3_^−^ incorporated from the esterification of hydroxyl groups using sulfuric acid [28,36]. The degree of esterification was determined by using Equation (3) in which the number of anionic sulfate groups per 100 anhydroglucose units (n_OSO3_) was calculated to be 2.82. This value was comparatively lower to other studies as Hamad and Hu were able to obtain a n_OSO3_ value of 4.7–6.7 using 64 wt% sulfuric acid with varying temperatures while Shafiei-Sabet et al. synthesized CNC with a 3.55 and 4.39 n_OSO3_ value using 64 wt% sulfuric acid with a different acid-to-pulp ratio [41,42].

In terms of m-CNC, not much difference in sulfur composition was observed, indicating no changes to the amount of OSO_3_^−^ on the surface of nanocellulose after the modification process. On the other hand, STAC (molecular formula of C_21_H_46_ClN) with a high content of carbon was grafted onto the surface of the nanocellulose. The increase in C to ~55 wt% and decrease in O composition to ~44 wt% could be observed for m-CNC as compared to CNC. This was because the stearyltrimethyl alkyl chain was successfully grafted onto the CNC surface and the surface oxygen was attached by the alkyl chain. By using Equation (4), the number of stearyltrimethylammoinum ions per 100 anhydroglucose units (n_STAC_) was found to be 2.86 which was the same with the n_OSO3_. This proved that the modification process was successful as all the OSO_3_^−^ were modified with STAC.

**Figure 4:**
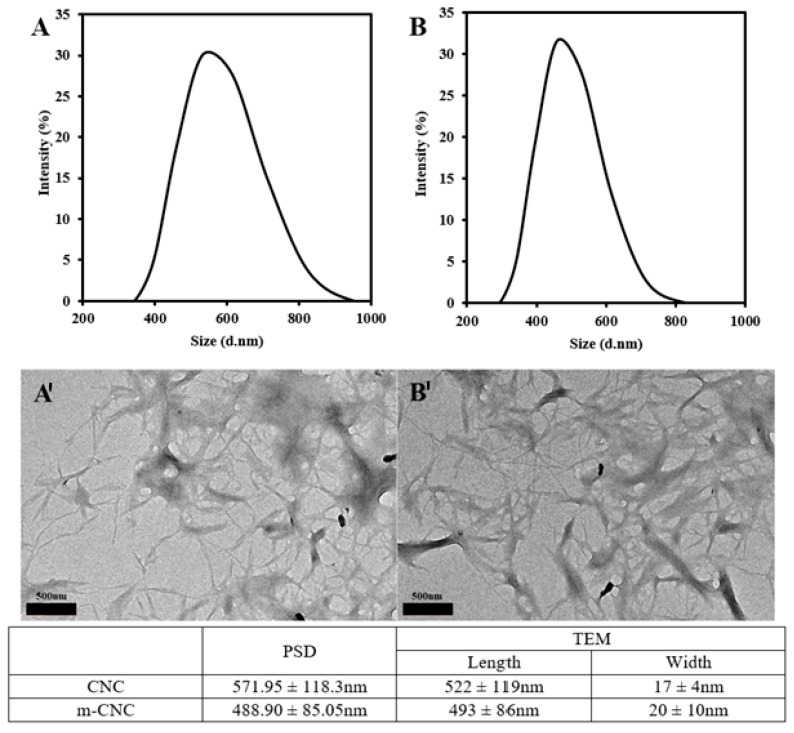
PSD ((**A**) CNC and (**B**) m-CNC) and TEM ((**A′**) CNC and (**B′**) m-CNC) microscopic images of both nanocelluloses with the scale bars representing 500 nm and their average particle size tabulated.

The particle sizes of both CNC and m-CNC were further studied by using the particle size distribution (PSD) and transmission electron microscopy (TEM) analysis (Figure 4). The PSD profile showed both samples had particle sizes within the range of nano-sizes, where CNC had an average size distribution of 571.95 ± 118.30 nm while m-CNC possessed an average size distribution of 488.90 ± 85.05 nm. The PSD were obtained using the dynamic light scattering technique which measured the diffusion of particles under Brownian motion. The technique is suitable for its use for the determination of colloidal particles like nanocellulose and the results obtained were in terms of the spherical hydrodynamic diameter. Meanwhile, TEM images indicated the presence of individual rod-like nanoparticles and agglomerated nanocellulose in a web-like arrangement with varying length and width. It was found that CNC showed an average length of 522 ± 119 nm and average width of 17 ± 4 nm. Meanwhile, m-CNC had an average length of 493 ± 86 nm and average width of 20 ± 10 nm. The average length of the nanocellulose calculated from the TEM images agreed with the results obtained from the PSD curve.

Analysis of variance (ANOVA) single factor test was performed on the length and width of both the nanocellulose particles to determine if there was a significant difference in their sizes with the results shown in the Supplementary Information (Table S1). The *p*-values obtained (0.29 for length and 0.22 for width) were higher than the significant level (α = 0.05) and did not indicate any variance observed between the length and width of CNC and m-CNC. We failed to reject the null hypothesis (H_o_) stating that there is no significant difference between the means of the length and width of the two groups. Thus, we could conclude that the modification process did not have a significant effect on the size of CNC.

### 2.2. Characterization of m-CNC-Stabilised Oil–Water Emulsion Gels

#### Colloidal Stability Profile of Oil–Water Emulsion Gels

**Figure 5:**
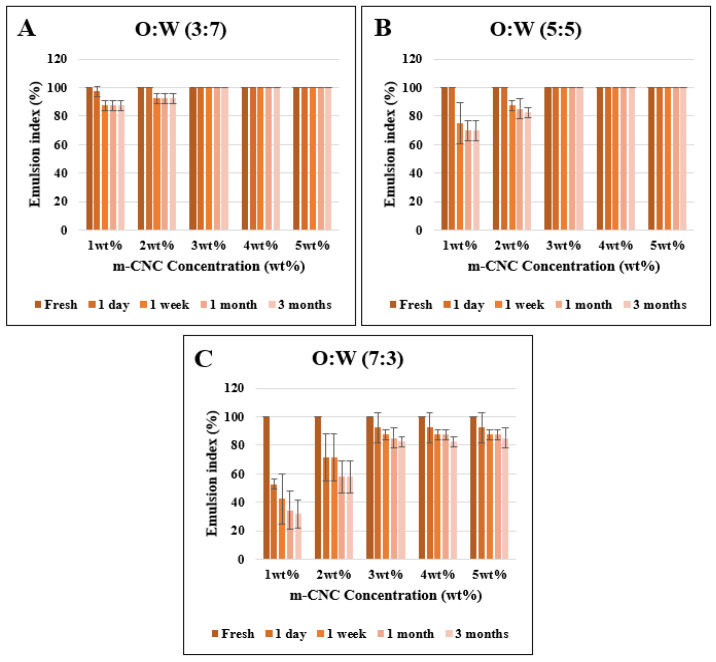
Emulsion index of the sample emulsions with 1–5 wt% concentration of modified nanocellulose with varying O:W ratios (**A**) 3:7, (**B**) 5:5, (**C**) 7:3.

In most applications, the stability of emulsion gels is crucial as any observed instability signifies the undesirable demulsification process, making it the most significant aspect to consider. A direct method to determine its stability was to determine the emulsion index (E.I) of each sample using Equation (5). Figure 5 displayed the E.I of m-CNC-stabilized emulsion gels with different m-CNC concentrations (1–5 wt%) and O:W ratios of 3:7, 5:5, and 7:3 for a storage period of 3 months. All the fresh emulsions were observed to be stable white cream gels with an E.I of 100%. However, the E.I of some samples decreased throughout the storage period. It was noted that all the fresh emulsion gels maintained a consistent pH level of 5. This pH value has been selected as it is the optimal condition to maintain the stability of the emulsion [43].

In the case of emulsion gels with O:W 3:7 and 5:5, a similar observation could be observed in their respective O:W ratio. The samples with 1 wt% and 2 wt% concentration of m-CNC exhibited creaming or/and sedimentation of emulsion after 1 week as phase separation of the cream emulsion or/and water could be observed clearly. On the other hand, emulsions stabilized with 3 wt% to 5 wt% concentration of m-CNC exhibited stable emulsion gels that lasted at least 3 months with no creaming observed with E.I values of 100%. This showed that concentration of m-CNC < 3 wt% was insufficient to form a stable colloidal network between oil and water, whereas a concentration of m-CNC ≥ 3 wt% could form a colloidal that could resist the creaming process for at least 3 months. The present observation was also in line with a study by Varanasi et al., in which a low concentration of CNC (<2 wt%) formed a partially stable canola oil and hexadecane emulsion (O:W ratio of 2:8), while concentrations of >3 wt% were able to form completely stable emulsions [44].

On the other hand, the fresh emulsion gels with O:W 7:3 were not stable as the sedimentation and phase separation of the cream emulsion and the oil were observed the next day. Their E.I decreased drastically after a week especially for the 7:3—1 wt% m-CNC and 7:3—2 wt% m-CNC, where they displayed an E.I ≤ 50% while samples 7:3—3 wt% m-CNC, 7:3—4 wt% m-CNC, and 7:3—5 wt% m-CNC were observed to have an E.I of ~80%. Not only that, but their E.I also decreased continuously throughout the 3-month period of observation.

Hence, the study indicated that emulsion gels with low m-CNC concentration were unable to form stable emulsions across all ranges of studied O:W ratios. The occurrence of creaming/sedimentation in the emulsion could be observed within a week of storage. This is due to insufficient m-CNC to establish an adsorption network between the oil–water interface [22,45]. However, increments of m-CNC (3–5 wt%) ensure sufficient particles to have better coverage of oil droplets, thus facilitating complete irreversible adsorption at the oil–water interface to fabricate stable emulsions [45]. In addition to that, the excess m-CNC particles were able to form a strong depletion colloidal network within the aqueous phase, restricting creaming or sedimentation of the emulsion, as observed for the O:W 3:7 and 5:5 sample emulsions [46].

**Figure 6:**
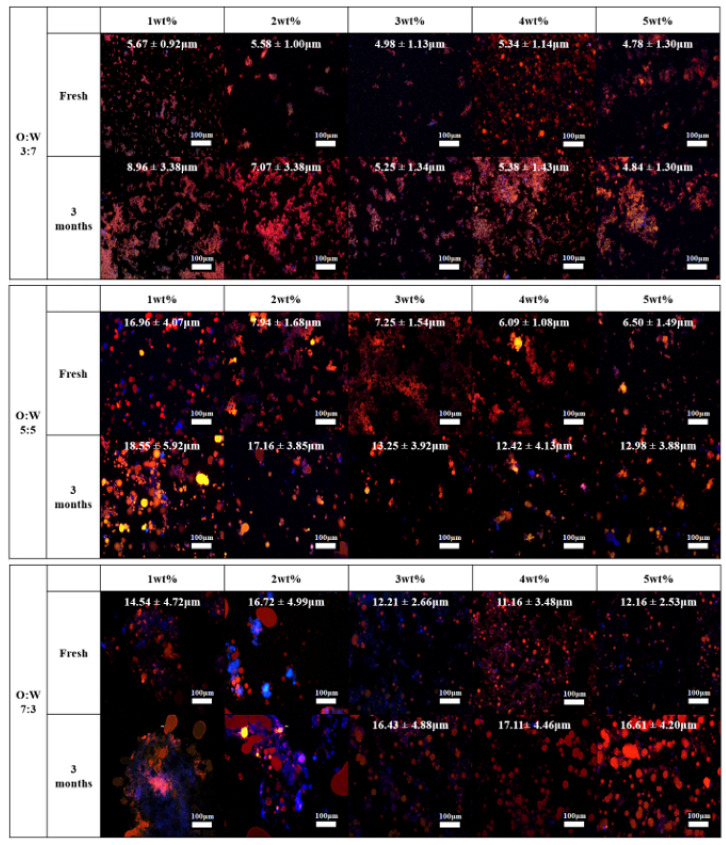
Fluorescent microscopic images of the fresh and after 3 months sample emulsion with their respective average oil droplet size and a scale bar of 100 μm included.

Fluorescence microscopy was used to investigate the microstructure and dispersion of oil droplets with m-CNC in emulsion gels. The microstructure of fresh sample emulsions was analyzed within 1–2 h after preparation. Figure 6 showed the microscopic fluorescent images of the samples at day 0 and day 90 (3 months), where the red color represents the oil phase, and the blue color represents the m-CNC. By using the ImageJ software application, the average oil droplet size was calculated with at least 50 oil droplets measured and tabulated as shown in Figure 6.

For the O:W 3:7 ratio, all the fresh sample emulsions displayed small oil droplets with an average diameter of ~5 μm. Flocs of oil droplets were also observed in all the samples instead of individual droplets which could be explained by the interfacial colloidal network of m-CNC. Excess amounts of m-CNC in the aqueous phase acted as a “bridge” and connected the oil droplets by interacting with the adsorbed m-CNC via hydrogen bonds [47]. After 3 months, flocculation of oil droplets was observed for all the samples as the flocs of droplets became larger. The average oil droplet sizes were shown to increase slightly from ~5 μm to ~7–9 μm for the samples of 3:7—1 wt% and 3:7—2 wt%. On the other hand, the droplet size for samples 3:7—3 wt%, 3:7—4 wt%, and 3:7—5 wt% showed negligible changes after a storage duration of 3 months.

The fresh emulsion gel (O:W ratio = 5:5) with different m-CNC concentrations showed homogenously distributed small oil droplets, except for the sample 5:5—1 wt% with uneven distribution with non-spherical droplets sized ~17μm. On the other hand, the emulsion samples 5:5—2 wt% to 5:5—5 wt% showed small oil droplets with an average droplet size of ~6–7 μm. The droplet size decreased with the increase in m-CNC concentration, as there were more solid particles to stabilize the oil–water interfaces [46]. After 3 months, the average oil droplet size was increased for all the samples as coalescence was observed for all the studied concentrations of m-CNC used. Both samples 5:5—1 wt% and 5:5—2 wt% displayed irregular and non-spherical oil droplets with an average oil droplet size of ~17–18 μm. Conversely, the microstructure of the 5:5—3 wt%, 5:5—4 wt%, and 5:5—5 wt% samples still displayed flocs of oil droplets after the storage period. However, their oil droplet sizes increased from ~6–7 μm to ~12–13 μm.

For the O:W 7:3 ratio, the emulsion microstructure of the 7:3—1 wt% and 7:3—2 wt% samples indicated an unstable emulsion formed. This was because of the observation of non-spherical and broken down oil droplets from the microscopic fluorescent images (Figure 6). On the other hand, the 7:3—3 wt%, 7:3—4 wt%, and 7:3—5 wt% samples showed to be able to form a stable emulsion with spherical oil droplets with an average size ranging around 11–12 μm. Flocculation of oil droplets, however, was not observed for the fresh samples unlike the samples with O:W ratios of 3:7 and 5:5. After 3 months, as expected, the emulsion microstructure for the 7:3—1 wt% and 7:3—2 wt% samples did not change much as blobs of broken down oil droplets were observed. As for the 7:3—3 wt%, 7:3—4 wt%, and 7:3—5 wt% samples, coalescence of oil droplets was discovered where the average oil droplet size increased to around 16–17 μm.

Based on results obtained from the microscopic fluorescent images, samples with 3 wt% of m-CNC rendered the most optimal concentration of emulsifier for all the studied O:W ratios. At a lower concentration of m-CNC (1 wt% and 2 wt%), the oil droplets formed were larger due to insufficient solid colloidal particles present to stabilize the droplets. The large droplets were also more prone to coalescence which then caused creaming or/and sedimentation of the emulsion to occur after 3 months of storage. However, there was no significant change in the size of droplets even though the concentration of m-CNC increased to 4 wt% or 5 wt%; therefore, 3 wt% of m-CNC was deemed the most optimal concentration of emulsifier for all the studied O:W ratios. A study by Tian et al. also reported similar findings when the concentration of β-cyclodextrin increased from 1% to 3%, this decreased the size of oil droplets but remained the same when its concentration increased from 3% to 5% [48]. Bai et al. also reported that the mean droplet diameter decreased when the CNC concentration increased from 0.05 to 0.75 wt%; however, the results did not show signification changes in particle size of ~1μm with a CNC concentration of 0.75 to 2.0 wt% [49]. They also described that at a high CNC concentration, there was excess CNC in the aqueous phase or a multilayer of CNC formed surrounding the oil droplets.

**Figure 7:**
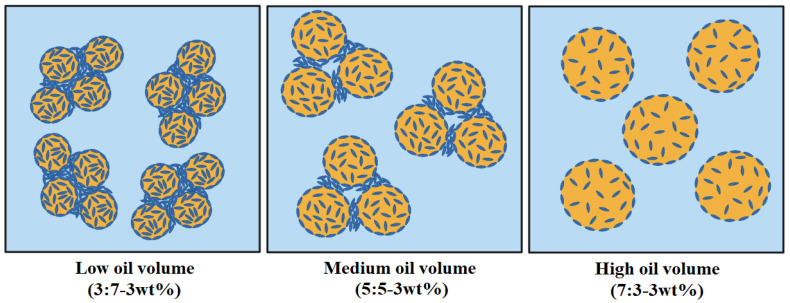
Schematic illustration of the microstructure of the emulsion at different O:W ratios. Created with BioRender.com.

By comparing the droplet size of the sample emulsion with the same concentration of m-CNC used across the O:W ratio, it was observed that an increase in the O:W ratio led to an increase in oil droplet size. This observation was consistent with all m-CNC concentrations used in this study, which proved that the O:W ratio of the emulsion is a key factor in affecting droplet size. At a tested concentration of 3 wt% m-CNC, the average oil droplet size was ~5 μm at O:W 3:7 and increased to ~7 μm, and ~12 μm for O:W = 5:5 and O:W = 7:3, respectively. After 3 months, their oil droplet size increased to ~5 μm, ~13 μm, and ~16 μm for the samples with O:W 3:7, 5:5, and 7:3, respectively. The increment of O:W ratio with higher concentrations of oil in the dispersed phase resulted in collision and coalescence (merging) between droplets, which formed larger sizes of oil droplets. Consequently, larger oil droplets reduced the interfacial area compared to smaller droplets, which affected the adsorption effectiveness of m-CNC at the interfaces, hindering its ability to stabilize all the droplets adequately [50]. This insufficiency in coverage of the oil droplets’ surfaces, promoting the recoalescence of droplets, the growth of the oil droplets, and eventual destabilization of the emulsion gel [43]. Furthermore, the enlargement of oil droplet size had a detrimental impact on the storage stability. This was due to the agglomeration of larger-sized droplets, leading to phase separation within the emulsion mixture. Consequently, a decrease in E.I from 100% to 80% was observed in the 3 wt% m-CNC samples with O:W ratios ranging from 3:7 to 7:3 [46]. We postulated that the microstructure of the emulsion differed with varying O:W ratios. A colloidal network was formed via hydrogen bonds from the excess m-CNC at a low O:W ratio emulsion while most of the m-CNC were adsorbed onto the oil droplets with not much left to form a colloidal network at a high O:W ratio emulsion. Figure 7 depicted a probable microstructure of the emulsion with varying oil-to-water ratios.

**Figure 8:**
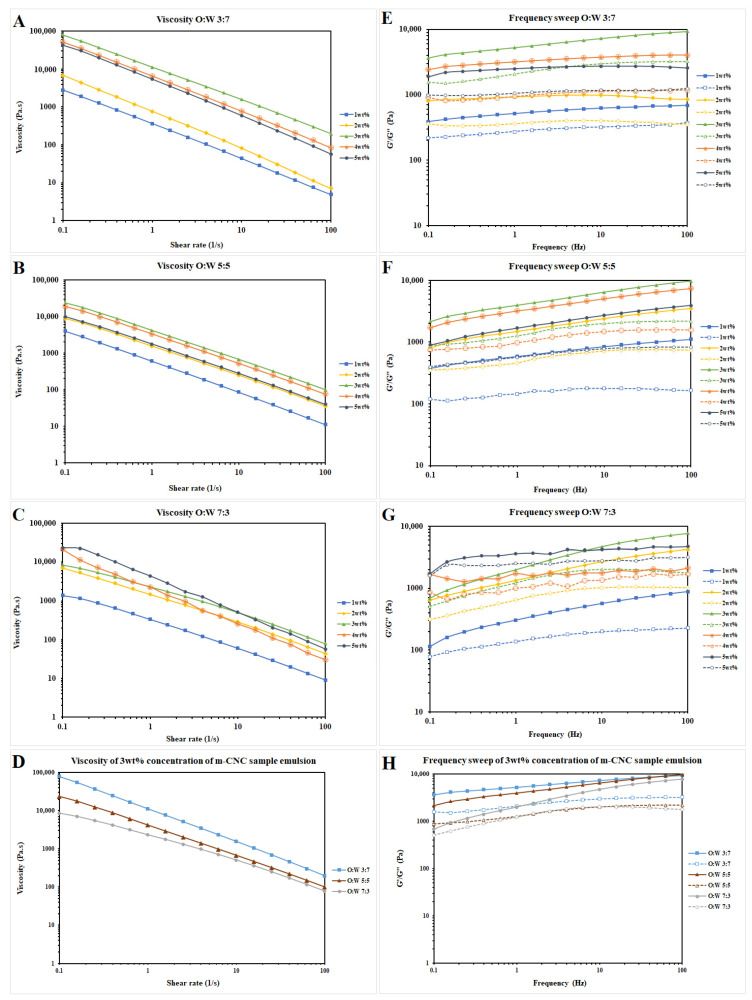
The viscosity versus shear rate curve of the emulsion samples with different concentrations of m-CNC (**A**–**C**) or O:W ratios (**D**). Storage modulus (closed symbols) and loss modulus (open symbols) of emulsion samples with different concentrations of m-CNC (**E**–**G**) or O:W ratios (**H**) as a function of angular frequency.

The rheological measurements of the emulsion gels allowed for an understanding of their physiochemical properties (e.g., stability and microstructure) under the influence of an external force. The rheology of fresh sample emulsions was analyzed within 1–2 h after preparation. The apparent viscosity and the frequency sweep curve of the m-CNC-stabilized emulsion gels were shown in Figure 8. The viscosity of the prepared samples (O:W ratio = 3:7, 5:5, and 7:3) decreased with the amplification of the shear rate, which indicated that the emulsions were non-Newtonian fluids with shear-thinning behaviors. Similar viscosity trends were observed for samples with different O:W ratios under increments of m-CNC concentration.

The storage modulus (G′) and loss modulus (G″) for all the emulsion gels were acquired by the frequency sweep test at an angular frequency ranging from 0.1 to 100 rad/s within the linear viscoelastic regime. All of the emulsions displayed a greater G′ than G″, which showed that the emulsions exhibited elastic behavior with the formation of emulsion gels (Figure 8E–G). The elastic behavior of the gel-like appearance of m-CNC-stabilized emulsions was due to the interactions between the m-CNC particles adsorbed on the adjacent oil droplets [51]. At the lower O:W ratios of 3:7 and 5:5, G′ increased from an emulsion sample with 1 wt% to 3 wt% of m-CNC, while it decreased from 3 wt% to 5 wt% of m-CNC with the trend of storage modulus from highest to lowest (3:7—3 wt%, 3:7—4 wt%, and 3:7—5 wt%, 3:7—2 wt%, and 3:7—1 wt%). This is because any excess m-CNC present would be dispersed within the continuous phase and formed colloidal networks encompassing the oil droplets. As the concentration of m-CNC increased (1 wt% to 3 wt%), the network structure became more compact and cohesive from the bridging phenomena of the nanocellulose particles which strengthened the gel-like viscoelastic network [45,52].

When the concentration of m-CNC increased to 4 wt% and 5 wt% from 3 wt%, a minor decrease in G′ was observed which indicated a slight deterioration of the emulsion structure. This can be attributed to the depletion flocculation effect caused by an excessive amount of m-CNC, which weakens the microstructure of the emulsion. However, the emulsion containing 3–5 wt% m-CNC exhibited high G′ due to strong interactions between m-CNC particles through van der Waals forces and hydrogen bonding. These interactions effectively restricted droplet movement and resisted phase separation [51].

However, the samples with an O:W 7:3 ratio did not show the same trend as with the O:W ratios of 3:7 and 5:5. The frequency sweep profile did not show a clear trend as the curves were intersecting with each other. Despite that, all the emulsions still exhibited elastic behavior even though the colloidal network was deemed to not be present in these samples. This was attributed to the m-CNC adsorbed onto the droplet interfaces interacting with each other as the frequency increased.

Amongst all the m-CNC concentrations, the emulsion with 3 wt% m-CNC rendered the most optimal concentration as it displayed the highest viscosity and G′ among their respective O:W ratios of emulsions, indicating a very stable emulsion gel was formed. However, as the O:W ratio increased at a constant concentration of m-CNC, the viscosity, both G′ and G″ were observed to decrease steadily. At a low O:W ratio of 3:7, it displayed the highest viscosity and G′ among all the samples in this study (Figure 8). This observation could be explained by the excessive m-CNC present in the aqueous phase of the emulsion mixture surrounding the oil droplets which strengthened the gel-like viscoelastic network that was able to resist phase separation of the emulsion during long storage. In addition, the excess m-CNC formed a dense colloidal network that resulted in flocs of oil droplets as observed in Figure 6.

As the O:W ratio increased to 5:5, the viscosity and G′ decreased. The increase in oil volume gave rise to an increase in the interfacial area of the dispersed phase and more m-CNC was needed to adsorb onto the interface between the two phases. The slight decrease in G′ indicated that the emulsion microstructure was not affected significantly. There was sufficient m-CNC in the aqueous phase to prevent phase separation for at least 3 months but was unable to prevent coalescence from occurring as the oil droplets were observed to increase.

At the highest O:W ratio of 7:3, the viscosity and G′ were observed to be the lowest among the three studied O:W ratios. Unlike the emulsion samples 3:7—3 wt% and 5:5—3 wt%, there were no colloidal networks formed in the sample 7:3—3 wt%, thus indicating that there were either limited or no m-CNC present in the aqueous phase. This was supported by the microscopic fluorescent images (Figure 6) as there was insufficient m-CNC to form flocs of oil droplets. The presence of low viscosity associated with low G′ facilitated the occurrence of coalescence and phase separation by enabling unrestricted movement and contact between the oil droplets. A study by Kinra and Pal used a wide range of concentrations of CNC (1.03–7.47 wt%) to form emulsions with varying concentrations of white mineral oil (10–70 wt%) [53]. They reported that at a fixed oil concentration, the increase in concentration of CNC increased the viscosity of the continuous phase which in turn increased the viscosity of the emulsion mixture. In addition, at a fixed concentration of CNC, the increase in oil concentration also increased the viscosity of the emulsion as more oil resisted the flow or movement of the oil droplets. This could be due to the highly negatively charged CNC being unable to form a colloidal network; thus, the different trend of viscosity was observed.

In short, the emulsion sample with 3 wt% of m-CNC and an O:W ratio of 3:7 showed to be the most stable emulsion with the smallest oil droplet size among all the samples. However, STAC had been studied to possess low acute toxicity like ocular and skin irritants at certain concentrations [54]. Despite that, it is considered to be non-irritating and safe to use in cosmetic formulations when its concentration is controlled. It is safe and does not pose a health risk under the concentration limits of 2.5% in rinse-off hair care products and 1.0% in leave-on hair care products [55]. On the other hand, as mentioned earlier, nanocelluloses extracted from lignocellulose biomass are low in toxicity and high in biocompatibility [11]. Therefore, the appropriateness of STAC-modified CNC for the cosmetic sector is applicable in relation to biocompatibility. However, further research and development are essential to establish the optimal safety concentration for product formulation.

## 3. Conclusions

In this study, STAC-modified CNC (m-CNC) was prepared from agricultural residues (SCB) as a value-upgraded nano-based Pickering stabilizer for circular economy aspects. The m-CNC was successfully synthesized as the FTIR spectroscopy and elemental analysis verified that the final product obtained was primarily made of cellulose compound and successfully introduced the trimethyl-alkyl chain onto the surface of the nanocellulose. The TGA analysis also showed that it had remarkable thermal properties which is useful in various high heat applications. TEM observation displayed bundles of uniform and rod-like-shaped nanoparticles with an average length of ~534 nm and average width of ~20 nm.

Subsequently, the potential of m-CNCs as Pickering stabilizers for oil–water emulsion gels was explored at varying concentrations (1–5 wt%) and oil-to-water (O:W) ratios (3:7, 5:5, 7:3) over a three-month period. The m-CNC with 3 wt% concentration promised the optimum stability of O:W emulsions by facilitating a strong colloidal network surrounding the oil droplets, resulting in a stable emulsion gel formed without occurrence of creaming/sedimentation. In addition, it also displayed a high viscosity and G′ that prevented or slowed down the coalescence process of small oil droplets for the selected duration of this study. To summarize, the utilization of STAC-modified CNC has shown promise as a sustainable Pickering stabilizer with diverse green and eco-friendly applications. This nano-emulsifier holds potential as a bio-based nonadditive across multiple fields dedicated to addressing environmental concerns, thereby reducing agricultural waste generation and environmental impact.

## 4. Materials and Methods

### 4.1. Materials

Sugarcane bagasse fiber, sodium hydroxide (NaOH), 50% hydrogen peroxide (H_2_O_2_), 97% sulfuric acid (H_2_SO_4_), stearyltrimethylammonium chloride (STAC), cooking oil (palm olein), Nile red, and Calcofluor white.

### 4.2. Methods

#### 4.2.1. Pre-Treatment of Sugarcane Bagasse

The SCB fibers were first dried in an oven, then grinded and passed through a 50-mesh size sieve. The grounded fibers were first pre-treated with 2 wt% NaOH solution at 70–80 °C under constant agitation for 4 h. This step was performed twice. The cellulose fibers were filtered and washed with distilled water until a constant pH of filtrate was obtained followed by drying in an oven with air circulation. Next, the cellulose fibers were bleached by suspending in a solution of 30% H_2_O_2_ and agitating for 4 h at 70–80 °C. The bleaching process was repeated until the fibers became pure white. The bleached fibers or CPC were filtered and washed with distilled water until a constant pH of filtrate was obtained then dried in an oven with air circulation.

#### 4.2.2. Synthesis of Modified Cellulose Nanocrystal

The CPC was subjected to acid hydrolysis to form cellulose nanocrystal (CNC). Briefly, the hydrolysis of cellulose was conducted using 48% (*w*/*w*) H_2_SO_4_ solution (1:10 g/mL) under mechanical stirring at 45 °C for 45 min. An excess amount of distilled water was then added to quench the hydrolysis reaction; subsequently, successive centrifugation was performed to withdraw the excess acid. The sediment was then gathered and dialyzed against distilled water until a neutral pH was obtained. After the dialysis process, the sample was sonicated in an ice bath before drying in an oven with air circulation.

Surface modification of CNC was conducted by using electrostatic attraction. In brief, 1 wt% of CNC suspension and 3 wt% of STAC solution were first prepared. Both solutions were heated to 60 °C before the CNC suspension was slowly added into the STAC solution. After all the CNC was added, the foamy mixture was stirred for 3 h with the temperature maintained at 60 °C and left to be stirred at room temperature overnight. The suspension was then centrifuged and dialyzed to remove any unbound STAC. Lastly, the m-CNC was dried in an oven with air circulation.

#### 4.2.3. Preparation of Pickering Emulsion Gels

In this emulsion gel study, distilled water and cooking oil were used as the polar and non-polar phases with different O:W ratios (3:7, 5:5, 7:3). Briefly, the cooking oil was added to the CNC aqueous suspension (concentration ranging from 1–5 wt% of the total mixture) in a 20 mL vial and ultrasonicated. Sodium azide was added into the emulsions to prevent the growth of microorganisms in order to investigate the storage stability of the emulsions at room temperature.

### 4.3. Characterization of SCB, CPC, CNC, and m-CNC

The total yield of the synthesized CNC was determined through the gravimetric method using the following equation below with W_raw_ being the dry weight of the raw material at each treatment stage, W_rawSCB_ being the dry weight of the raw SCB, and W_product_ being the dry weight of the product obtained. Each measurement was performed in a triplicate fashion and the results were expressed as the average value ± its standard deviation.

(1) $$Yield\left(\%\right)=\frac{W_{product}}{W_{raw}}\times 100\%$$

(2) $$Yield\left(\%\right)=\frac{W_{product}}{W_{rawSCB}}\times 100\%$$

FTIR analysis was carried out to determine the chemical compositions and functional groups of the samples. The FTIR spectra of the samples were obtained from the Fourier transform infrared spectrometer (Perkin Elmer, SPECTRUM 400, Waltham, MA, USA) using the transmittance mode under a range of wavenumbers from 4000 to 600 cm^−1^ at a spectral resolution of 4 cm^−1^. TGA analysis was used to study the thermal stability as well as the degradation temperature of the samples using Mettler Toledo TGA/SDTA 851e (Columbus, OH, USA). The samples were placed in a crucible and heated from 25 to 600 °C under a nitrogen environment with a heating rate of 10 °C min^−1^. The surface morphology of each sample was investigated using a field emission scanning electron microscope (FEI QUANTA FEG 450, Brno, Czech Republic) at an accelerating voltage of 7 kV. The samples were mounted onto aluminum stubs with the help of double-sided carbon tapes and coated in gold using a vacuum sputter coater. After that, the elemental composition of each sample was analyzed using Perkin Elmer CHNS/O 2400 Series II (Shelton, CT, USA) and EDX spectroscopy (Waltham, MA, USA). The number of anionic sulfate groups (n_OSO3_) and stearyltrimethylammoinum ions (n_STAC_) per 100 anhydroglucose units were calculated from the equations as shown below as reported in a study by Hamad and Hu [41].

(3) $$S\left(wt\%\right)=\frac{n_{SO3}S\times 100}{6C+10H+5O+n_{SO3}(S+3O)}\times 100\%$$

(4) $$N\left(wt\%\right)=\frac{n_{STAC}N\times 100}{6C+10H+8O+S+n_{STAC}(21C+46H+N)}\times 100\%$$

The PSD of the synthesized CNC and m-CNC were measured with the dynamic light scattering method using a Malvern Zetasizer Nano ZS instrument (Worcestershire, England). Firstly, 5 mL of CNC dispersion was sonicated for 5 min in an ultrasonic bath before transferring into a folded capillary cell for the analysis. The morphology and topography of the synthesized CNC were observed using high resolution transmission electron microscopy (JEOL, JEM-2100F, Tokyo, Japan) at an accelerating voltage of 200 kV. Then, 1 mL of CNC dispersion was deposited on a carbon-coated copper grid and left to dry in a well-ventilated area prior to analysis. Digital image analysis (Version: ImageJ 1.53e) was then utilized to calculate the average length of nanocellulose by analyzing at least 30 readings. The data obtained were subjected to ANOVA single factor test at a 5% significance level (α = 0.05) (Table S1).

### 4.4. Characterization of Pickering Emulsion Gels

The prepared emulsion gels were labelled as the O:W ratio followed by the concentration of m-CNC as the Pickering stabilizer. For example, an emulsion gel with an O:W 3:7 ratio and 1 wt% of m-CNC was labelled as 3:7-1 wt%. The stability of the emulsion was measured by observing the emulsion volume of each Pickering emulsion sample. The samples were kept at room temperature and their images were taken each day from day 0 until 3 months. The emulsion volume of the samples was examined by calculating the E.I using the equation as follows:

(5) $$E.I=\frac{H_{E}}{H_{T}}\times 100\%$$

where H_E_ is the height of the emulsion layer, while H_T_ is the height of the total emulsion. The value of H_E_ and H_T_ were measured through visual observation of the images of each sample. Each measurement was done in a triplicate fashion with their standard deviation shown as error bars in the figure.

The microstructure of the emulsion layer was observed using the fluorescence microscope (Leica, Leica DMI6000B, Tokyo, Japan). The oil phase was dyed using Nile red solution while the modified CNC on the other hand was dyed with Calcofluor white before observation. The average oil droplet size was reported as D [2, 0] by measuring diameters of more than 50 droplets and expressed as the average value ± its standard deviation. Rheology of the gel-like emulsions was studied using a rheometer (TA Instruments, DHR-2, New Castle, DE, USA). The amplitude sweep test was conducted from 0.01 to 100% angular strain at a constant frequency of 6.28 rad s^−1^ to determine the linear viscoelastic region of each sample. The frequency sweep test was performed with an angular frequency range of 0.1–100 rad s^−1^ at a constant strain of 0.1% and the viscosity was measured at a shear rate of 0.1–100%. The analysis of all fresh sample emulsions was examined within 1–2 h after preparation.

## Figures and Tables

**Table 1:** Percentage yield of product from each respective process and raw SCB.

Samples	Percentage Yield from Process ^a^ *	Percentage Yield from SCB ^b^ *
CPC (After pre-treatment)	41.01 ± 1.48%	41.01 ± 1.48%
CNC (After acid hydrolysis)	37.81 ± 1.44%	15.51 ± 3.93%

^a^ The sample yield (%) was determined based on the total mass product of each stage of treatment (Equation (1)). ^b^ The sample yield (%) was determined based on the total mass raw SCB (Equation (2)). * All calculations were obtained from an average of three readings and recorded as (mean value ± standard deviation).

**Table 2:** Elemental composition of CPC, CNC, and m-CNC from EDX analysis.

CPC			CNC			m-CNC		
Element	Wt%	Atomic %	Element	Wt%	Atomic %	Element	Wt%	Atomic %
C	50.28	57.39	C	49.52	56.78	C	55.28	62.36
O	49.72	42.61	O	49.96	42.98	O	44.18	37.41
S	0.00	0.00	S	0.55	0.24	S	0.54	0.23
N	0.00	-	N	0.00	-	N	0.16	-

## Data Availability

Not applicable.

## Supplementary Materials

The following supporting information can be downloaded at: https://www.mdpi.com/article/10.3390/gels9090734/s1, Table S1. ANOVA single factor analysis for nanocellulose particle size.
