# Supplementary material for: Surface Functionalization of Sugarcane-Bagasse-Derived Cellulose Nanocrystal for Pickering Emulsion Gel: Microstructural Properties and Stability Efficiency
Source: Gels. 2023 Sep 9;9(9):734. doi: 10.3390/gels9090734 (PMC10528861; doi:10.3390/gels9090734)
Supplement: Supplementary file 1 [file gels-09-00734-s001.zip › gels-2526004-supplementary.pdf]

Table S1. ANOVA single factor analysis for nanocellulose particle sizes

Length (nm)	SS	df	MS	F	P-value	F crit
Between Groups	12441.6	1	12441.6	1.15632	0.286683	4.006873
Within Groups	624060	58	10759.66			
Total	636501.6	59				
**Width (nm)**	**SS**	**df**	**MS**	**F**	**P-value**	**F crit**
Between Groups	91.26667	1	91.26667	1.512995	0.223649	4.006873
Within Groups	3498.667	58	60.32184			
Total	3589.933	59				
